# Antibody-drug conjugates as immuno-oncology agents in colorectal cancer: targets, payloads, and therapeutic synergies

**DOI:** 10.3389/fimmu.2025.1678907

**Published:** 2025-11-03

**Authors:** Yihan Wang, Kai Lu, Yang Xu, Shengshan Xu, Hongyu Chu, Xuedong Fang

**Affiliations:** ^1^ ChinaJapan Union Hospital of Jilin University, Changchun, China; ^2^ Department of Thoracic Surgery, Jiangmen Central Hospital, Jiangmen, Guangdong, China; ^3^ Department of Gastrointestinal and Colorectal Surgery, ChinaJapan Union Hospital of Jilin University, Changchun, China

**Keywords:** colorectal cancer, microsatellite stable, antibody-drug conjugate, immunoconjugate, immuno-oncology

## Abstract

Colorectal cancer (CRC), particularly the immunologically “cold” microsatellite-stable (MSS) subtype, remains profoundly resistant to immune checkpoint inhibitors. Antibody-drug conjugates (ADCs) are rapidly emerging as a transformative therapeutic modality poised to overcome this challenge. This review reframes ADCs beyond their role as targeted cytotoxics, repositioning them as sophisticated immuno-oncology agents. The central thesis is that by strategically selecting payloads such as topoisomerase inhibitors or auristatins, modern ADCs can induce immunogenic cell death (ICD) or pyroptosis. This mechanism effectively functions as an *in situ* vaccine, transforming the tumor microenvironment from “cold” to “hot” by promoting dendritic cell activation and T-cell infiltration. We provide a comprehensive overview of the ADC landscape, examining key targets on bulk tumor cells (CEACAM5, HER2), cancer stem cells (LGR5, GPR56), and stromal components. We conclude that the future of ADCs in CRC lies in their rational application as immune-priming agents, creating powerful synergies in combination with checkpoint inhibitors to break therapeutic resistance and durably improve patient outcomes.

## 1 Introduction

Colorectal cancer (CRC) remains a major global health burden, ranking as the third most frequently diagnosed cancer and the second leading cause of cancer-related mortality worldwide (1), with over 1.9 million new cases and approximately 935,000 deaths reported in 2020 (2). While standard treatments, including chemotherapy and targeted agents, form the backbone of systemic therapy, their efficacy is often curtailed by tumor heterogeneity and acquired resistance (3). More critically, the transformative success of immune checkpoint inhibitors (ICIs) has not extended to the majority of CRC patients, whose tumors are microsatellite-stable (MSS). These immunologically “cold” tumors are characterized by a non-inflamed tumor microenvironment (TME) that lacks T-cell infiltration, rendering them profoundly resistant to current immunotherapies (4–6). This reality underscores an urgent need for novel therapeutic strategies capable of inducing a robust, *de novo* anti-tumor immune response.

Antibody-drug conjugates (ADCs) have emerged as a powerful therapeutic modality uniquely positioned to bridge the gap between targeted chemotherapy and immunotherapy (7). An ADC consists of a monoclonal antibody targeting a tumor-associated antigen, a highly potent cytotoxic payload, and a chemical linker. However, beyond their original design as “magic bullets,” modern ADCs are increasingly engineered as sophisticated immuno-oncology agents. This advanced function is achieved through the strategic selection of payloads-such as topoisomerase inhibitors or microtubule inhibitors-that are potent inducers of immunogenic cell death (ICD) or other pro-inflammatory pathways like pyroptosis. By forcing cancer cells to die in an immunologically active manner, these ADCs can effectively transform the tumor into an *in situ* vaccine, triggering the release of danger signals and tumor antigens that awaken the immune system (8). The landmark approvals of agents like trastuzumab deruxtecan and sacituzumab govitecan have validated this dual-action approach, demonstrating that ADCs can produce durable responses even in heavily pretreated patient populations (9–11).

In CRC, this dual mechanism holds immense promise for overcoming the intrinsic resistance of MSS tumors. By delivering an immunogenic payload directly to tumor cells, ADCs can initiate an inflammatory cascade, remodel the immunosuppressive TME, and prime a T-cell-mediated immune attack. This provides a compelling rationale for combining ADCs with checkpoint inhibitors to create potent synergistic effects. The expanding landscape of ADC targets in CRC now includes not only antigens on bulk tumor cells (CEACAM5, HER2), but also those on cancer stem cells (LGR5) and critical stromal components (CEACAM6), offering multiple avenues to dismantle the tumor ecosystem.

This review provides a comprehensive overview of the ADC landscape in CRC, framed through an immuno-oncological perspective. We will dissect their molecular architecture and mechanisms of action, with a special focus on their ability to modulate the immune system. We will then survey the key therapeutic targets-from established to emerging-and discuss how they can be leveraged as specific gateways for delivering immunogenic payloads. Moreover, we will explore how ADCs are poised to reshape CRC treatment paradigms, not as standalone agents, but as cornerstone therapies in the next generation of rational, immune-based combination strategies.

### 1.1 Literature search strategy

A systematic literature search was performed using PubMed/MEDLINE, Embase, Web of Science, and ClinicalTrials.gov databases through September 2025. The search strategy combined MeSH terms and keywords with Boolean operators, encompassing: (1) disease terms (“colorectal cancer” OR “colorectal neoplasms”[MeSH] OR “CRC” OR “microsatellite stable”); (2) intervention terms (“antibody-drug conjugate” OR “immunoconjugate” OR “ADC”); and (3) mechanism terms (“immuno-oncology” OR “immunogenic cell death” OR “ICD” OR “pyroptosis” OR “tumor microenvironment” OR “TME” OR “combination therapy”). Additional targeted searches were conducted for specific ADC targets and agents (e.g., CEACAM5, HER2, trastuzumab deruxtecan, LGR5). Besides, peer-reviewed original research articles, clinical trials, authoritative reviews, and relevant conference abstracts from major oncology meetings (ASCO, ESMO) published in English were included. Studies were selected based on their relevance to ADCs as immuno-oncology agents in CRC. Case reports without mechanistic insights and editorials were excluded. Reference lists of included articles were manually screened to identify additional relevant publications.

## 2 Structure, mechanism of action and immune regulation of ADC

### 2.1 Structure of ADC

**Figure 1:**
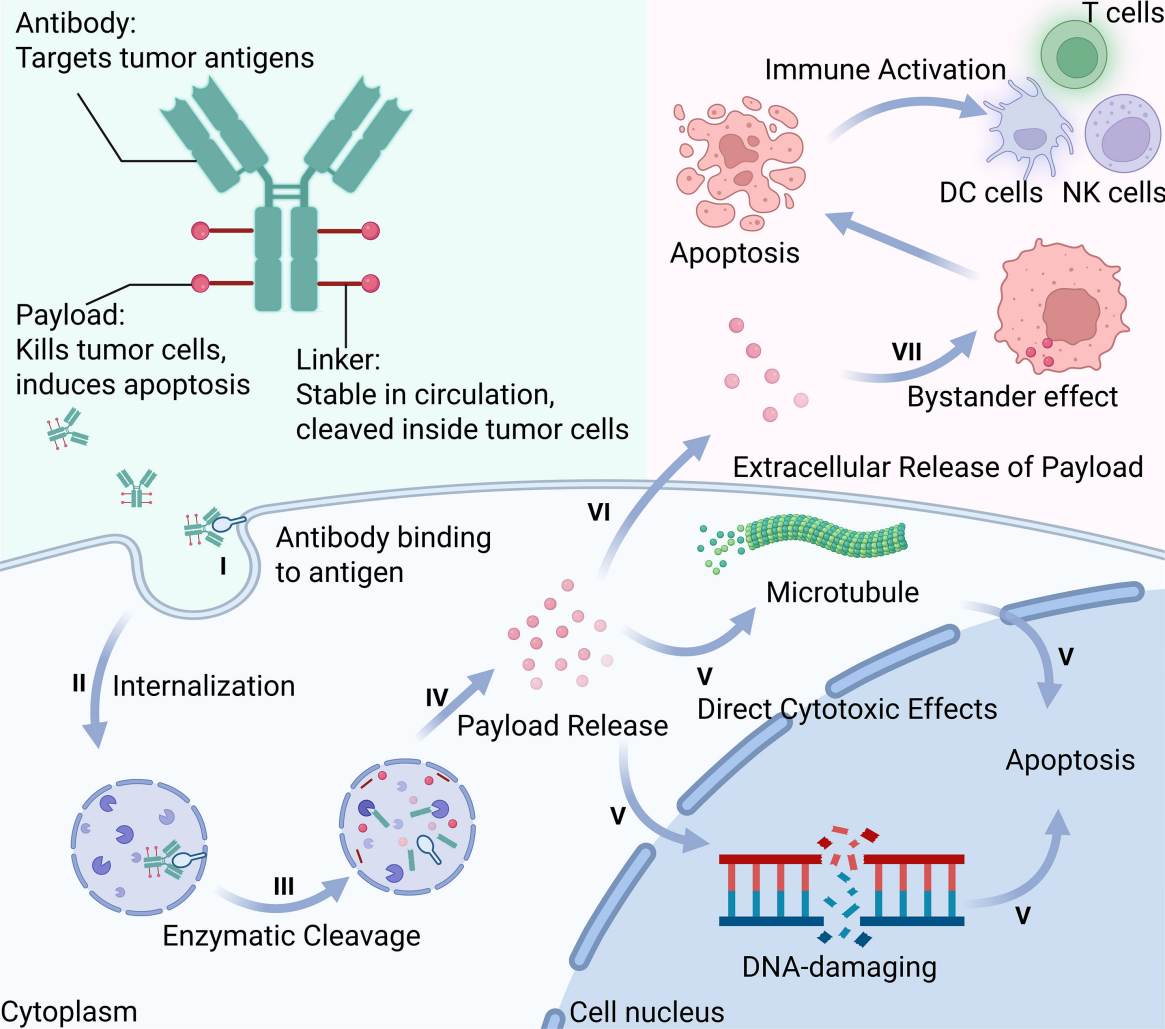
Schematic illustration of the seven-step mechanism of action of antibody-drug conjugates (ADCs): I. Antibody Binding to Antigen-The antibody specifically binds to tumor-associated surface antigens; II. Internalization-The ADC-antigen complex is internalized via receptor-mediated endocytosis; III. Enzymatic Cleavage-The linker is cleaved by intracellular enzymes within endosomes or lysosomes; IV. Payload Release-The cytotoxic payload is released into the cytoplasm or nucleus; V. Direct Cytotoxic Effects-The payload disrupts essential cellular functions by targeting DNA or microtubules; VI. Extracellular Release of Payload-A portion of the payload may exit the cell through lysis or active efflux; VII. Bystander Effect-The released payload kills neighboring tumor cells lacking target antigen. This figure was created using BioRender.com.

ADCs comprise three core elements: a tumor-targeting monoclonal antibody, a chemical linker, and a cytotoxic payload. Therapeutic efficacy depends on optimal integration of target antigen selection, antibody format, linker stability, payload potency, and conjugation chemistry (**Figure 1**). Ideal target antigens exhibit high tumor-specific expression (>10^5^ copies/cell), minimal normal tissue expression, efficient internalization, and limited shedding (12, 13). In CRC, promising targets include HER2, TROP2, CEACAM5, and mesothelin, each with distinct expression patterns and internalization kinetics.

The antibody backbone, typically humanized IgG1, provides targeting specificity and immune effector functions. IgG1 predominates (used in T-DXd, T-DM1, sacituzumab govitecan) due to its long half-life (~21 days) and ability to mediate Antibody-Dependent Cell-mediated Cytotoxicity (ADCC) and Complement-Dependent Cytotoxicity (CDC) (14–16). IgG4 variants reduce immune activation when inflammatory toxicity is a concern, though this may compromise immune-mediated effects (17). Antibody engineering, including Fc modifications, can optimize pharmacokinetics and safety profiles (18).

Linker chemistry critically balances plasma stability with intracellular payload release. Cleavable linkers (protease-sensitive valine-citrulline, pH-sensitive hydrazones, glutathione-reducible disulfides) enable controlled payload release and bystander effects (19, 20). Non-cleavable linkers require complete antibody degradation, offering superior stability but limited bystander activity (21, 22). Modern site-specific conjugation using engineered cysteines, enzymatic methods, or glycan remodeling yields homogeneous products with optimized drug-to-antibody ratios (DAR) (23–26). While traditional ADCs employed DAR 2-4, next-generation ADCs like T-DXd achieve DAR ~8 through improved linker-payload design (27, 28).

Payload selection determines cytotoxicity, resistance profiles, and immunogenic potential. Payloads with IC50 values in picomolar to low nanomolar range are categorized by mechanism: microtubule inhibitors, DNA-damaging agents (PBD dimers), and topoisomerase I inhibitors (DXd) (29).Membrane-permeable payloads like MMAE enable bystander killing of antigen-negative cells, while charged molecules (MMAF) remain cell-confined (30–33) DNA-damaging agents offer distinct advantages: topoisomerase I inhibitors provide broader therapeutic windows enabling higher DAR and reduced MDR1-mediated resistance (34, 35). PBD dimers show extreme potency but hepatotoxicity concerns in CRC, DXd-based ADCs demonstrate particular promise given their superior immunogenic cell death induction and favorable safety profiles, making them ideal for immunotherapy combinations (36). Payload hydrophobicity influences ADC stability-hydrophilic modifications enable higher drug loading without compromising pharmacokinetics.

### 2.2 Mechanisms of action

#### 2.2.1 Targeted delivery and intracellular processing

ADC action initiates through high-affinity antigen binding, embodying the “magic bullet” concept (37). Beyond targeted delivery, some ADCs exert direct antitumor effects through receptor signaling interference without payload release (38). Following receptor-mediated endocytosis primarily via clathrin-dependent pathways (39), ADCs traffic through endolysosomal systems where pH changes and proteolytic enzymes trigger linker cleavage (40). The intracellular fate depends on linker design-acid-labile bonds dissociate at lysosomal pH, protease-cleavable peptides undergo enzymatic hydrolysis, while disulfide bonds reduce in cytoplasmic environments. Once released, microtubule inhibitors disrupt mitotic spindle formation (41), while DNA-damaging agents induce strand breaks and apoptosis, with some payloads additionally triggering immunogenic cell death pathways (34, 42).

#### 2.2.2 Bystander effect as immune amplifier

The bystander effect represents a critical mechanism for overcoming CRC heterogeneity (43). This phenomenon depends on payload diffusion from targeted cells to eliminate adjacent antigen-negative populations (32, 44). Novel strategies like caspase-3-cleavable linkers create amplification loops where apoptosis triggers extracellular ADC cleavage (45). Importantly, bystander-mediated killing extends beyond cytotoxicity-dying cells release DAMPs and tumor antigens, spatially amplifying signals throughout the TME. This converts immunologically “cold” regions into “hot” zones, enhancing dendritic cell recruitment and T-cell priming across the entire tumor mass. The membrane permeability of payloads determines the extent of this effect: hydrophobic molecules like DXd and SN-38 freely traverse cell membranes to eliminate neighboring cells, while charged payloads remain confined. This spatial extension of both cytotoxic and immunogenic effects transforms heterogeneous tumors into more uniformly targeted tissues, creating a foundation for the comprehensive immune response.

### 2.3 Immunomodulatory mechanisms of antibody-drug conjugates

Beyond direct cytotoxicity, ADCs demonstrate capacity to modulate antitumor immune responses through multiple mechanisms engaging various stages of the cancer-immunity cycle (46).

#### 2.3.1 Payload-induced immunogenic cell death

Colorectal cancer presents distinct immunological landscapes across molecular subtypes that shape therapeutic opportunities for ADC development. The predominant microsatellite stable (MSS) phenotype, comprising 85% of cases, exhibits an immunologically inert tumor microenvironment characterized by sparse lymphocyte infiltration and minimal neoantigen presentation, rendering these tumors refractory to checkpoint blockade. In contrast, the microsatellite instability-high (MSI-H) subset demonstrates robust immune infiltration yet develops alternative resistance mechanisms. This dichotomy underscores the need for ADCs capable of immunological reprogramming, particularly in converting immunologically “cold” MSS tumors into inflamed, treatment-responsive phenotypes.

Cytotoxic payloads transform tumor cell death into immunological priming events through DNA damage or microtubule disruption. This process generates three immunogenic signals: damage-associated molecular patterns (DAMPs) including ATP as chemotactic “find me” signals, HMGB1-TLR4 interactions driving DC maturation, and surface calreticulin serving as phagocytic “eat me” signals; enhanced tumor antigen cross-presentation; and pro-inflammatory cytokine release (IFN-γ, IL-1β, IL-6) recruiting effector lymphocytes (36, 47–49). Furthermore, cytokines and chemokines released by dying tumor cells following ADC treatment can recruit and activate various immune cells, including macrophages and natural killer (NK) cells, thereby further promoting antitumor responses (50).

Different payload classes exhibit distinct ICD profiles. DNA-damaging agents, particularly deruxtecan and camptothecin derivatives, induce robust DAMP release and type I interferon responses. In an *in vivo* study, T-DXd treatment resulted in increased expression of PD-L1 and MHC class I molecules on cancer cells (35).Pyrrolobenzodiazepine dimers generate more limited ICD despite high potency. Among microtubule inhibitors, maytansinoids (DM1/DM4) and auristatins (MMAE/MMAF) trigger ICD through mitotic, with membrane-permeable MMAE enabling bystander ICD amplification (39, 40). Interestingly, microtubule-depolymerizing payloads (such as vinca alkaloids) have been shown to induce dendritic cell maturation, while the same property has not been observed with microtubule-stabilizing agents (such as taxanes) (51). Alternative death modalities-pyroptosis through gasdermin pores, ferroptosis via lipid peroxidation, and necroptosis through RIPK3/MLKL-provide additional inflammatory signals that sustain immune activation.

#### 2.3.2 Antibody-mediated immune effector functions

The Fc domain mediates payload-independent immunity through antibody-dependent cell-mediated cytotoxicity (ADCC), antibody-dependent cellular phagocytosis (ADCP), and CDC mechanisms. ADCC involves IgG1-based ADCs engaging FcγRIIIA receptors on natural killer cells, triggering cytotoxic granule release for target cell lysis. For instance, trastuzumab-based ADCs demonstrate particularly robust NK cell-mediated killing of HER2-positive tumor cells. ADCP occurs when the Fc region binds FcγRI/II receptors on macrophages, promoting tumor cell engulfment and destruction, with studies showing T-DM1 enhances macrophage phagocytic activity in preclinical models. CDC is initiated when C1q binds to clustered Fc domains, activating the complement cascade that both forms membrane attack complexes for direct cell lysis and generates anaphylatoxins (C3a/C5a) to recruit and activate myeloid cells. Beyond these primary mechanisms, ADC-antigen immune complexes enhance cross-presentation through FcγR-dependent uptake by antigen-presenting cells (APCs), broadening the immune response. These effector functions remain operational against cells with defective endocytosis, ensuring therapeutic activity across heterogeneous tumor populations. This antibody-driven inflammation synergizes with payload-induced ICD, creating multiple complementary immune activation pathways that maintain efficacy despite variable antigen expression or payload resistance.

#### 2.3.3 TME reprogramming and therapeutic synergies

ADCs orchestrate comprehensive immune landscape remodeling. Previous studies have demonstrated ADC-mediated immune modulation across multiple cell populations: T-DM1 treatment polarizes tumor-associated macrophages from M2 to M1 phenotypes through TLR4/SCARA5 modulation, while sacituzumab govitecan enhances macrophage phagocytic activity (52). NK cells undergo dual activation via Fc-dependent mechanisms and stress ligand recognition, with trastuzumab-based ADCs showing particularly robust NK cell engagement (53, 54). The lymphocyte compartment experiences selective modulation-ADC treatment induces chemokine gradients (CXCL9, CCL3/4) that recruit effector T cells while regulatory populations undergo preferential depletion, as observed in T-DXd-treated tumors where Treg/CD8^+^ ratios shift favorably toward antitumor responses (55–57).

This comprehensive TME reprogramming creates strategic therapeutic synergies, particularly with immune checkpoint inhibitors. In colorectal cancer, the differential TME landscapes between MSI-H (~15%, immune-infiltrated) and MSS (~85%, immune-excluded) tumors dictate therapeutic responses (58). ADC-mediated TME reprogramming is especially transformative for “cold” MSS tumors: barrier disruption and immune cell recruitment, combined with checkpoint upregulation (PD-L1/CTLA-4), convert these ICI-resistant tumors into responsive phenotypes. This TME reprogramming immune activation has shown clinical validation, with a HER2-positive/RAS-mutant/MSS case achieving >10 months PFS using T-DXd plus serplulimab (58). Novel combinations advancing through trials include disitamab vedotin with tislelizumab (NCT05493683) and SBT6050-a HER2-targeting ADC carrying TLR8 agonist payload that directly bridges cytotoxic and immunostimulatory mechanisms (NCT04460456) (59). These multi-faceted immunomodulatory effects position ADCs as crucial enablers for converting ICI-resistant MSS tumors into treatment-responsive phenotypes.

## 3 Major ADC targets and drugs

**Figure 2:**
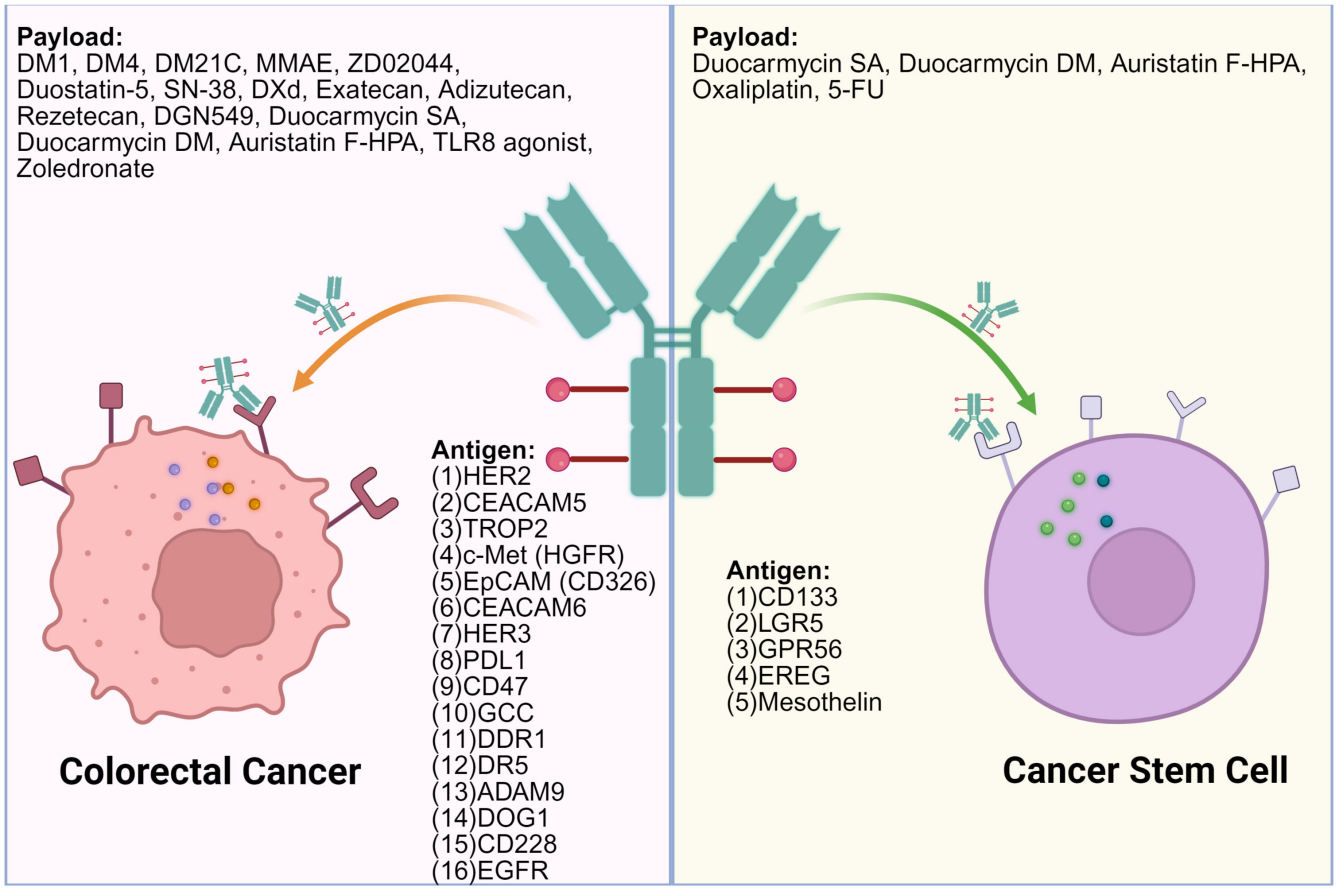
Surface targets on colorectal cancer cells and colorectal cancer stem cells and the associated ADC payloads. OXA, Oxaliplatin; MMAE, Monomethyl Auristatin E; PNU159682, PNU-159682; Duocarmycin SA, Duocarmycin Se-A; Tubulysin, Tubulysin; DM4, Maytansinoid DM4; SN-38, 7-Ethyl-10-hydroxycamptothecin; RNase A, Ribonuclease A; DM1, Maytansinoid DM1; DXd, Deruxtecan; DGN549, DGN549; DM21C, Maytansinoid DM21C; PMMAE, Polymeric Monomethyl Auristatin E; IGN, Indolinobenzodiazepine DNA-alkylating agent. This figure was created using BioRender.com.

ADCs exert antitumor effects by targeting surface antigens and delivering cytotoxic agents. In CRC, these targets can be categorized into those expressed on tumor cell surfaces and those found on cancer stem-like cells (CSCs). Tumor cell-associated targets allow broad cytotoxic coverage, while stem cell-associated targets focus on eliminating therapy-resistant populations. The following sections summarize the major ADC targets in CRC and their corresponding drug candidates under development or clinical evaluation (**Table 1**). The distribution of targets and associated payloads across different cell types is illustrated in **Figure 2**.

**Table 1:** Ongoing and completed clinical trials of antibody-drug conjugates in colorectal cancer.

NCT	Drug (ADC)	Phase	N	Key results	Target	Status
NCT03384940	T-DXd (Trastuzumab deruxtecan)	II	86	HER2^+^: ORR 45.3%, mPFS 6.9 mo, mOS 15.5 mo; HER2^-^: ORR 0%	HER2	Completed
NCT04744831	T-DXd	II	122	5.4 mg/kg: ORR 37.8%, mPFS 5.8 mo, mOS 13.4 mo; 6.4 mg/kg: ORR 27.5%, mPFS 5.5 mo	HER2	Completed
NCT03602079	A166	I/II	49	Phase I/II completed; no efficacy data reported	HER2	Completed
NCT03821233	ZW49	I	112	Phase I completed; awaiting efficacy data	HER2	Completed
NCT04513223	SHR-A1811	I	101	Ongoing study in GC/GEJ and CRC	HER2	Active, not recruiting
NCT04479436	U3-1402 (Patritumab deruxtecan)	I/II	–	–	HER3	Terminated
NCT05029882	ABBV-400 (Telisotuzumab adizutecan)	I	122	2.4 mg/kg: ORR 15%, mPFS 5.3 mo; 3.0 mg/kg: ORR 20%, mPFS 4.5 mo	c-Met	Active
NCT05464030	M9140 (Precem-TcT)	I	40	ORR 7.5%, mPFS 5.9 mo; ≥2.4 mg/kg: mPFS 6.7 mo	CEACAM5	Active
NCT02187848	SAR408701 (Tusamitamab ravtansine)	I/II	43	ORR 45%, DCR 83%	CEACAM5	Completed
NCT01605318	IMMU-130 (Labetuzumab govitecan)	I/II	–	Safety, ORR pending	CEACAM5	Active
NCT06265688	CX-2051	I	25	Expansion doses (n=18): ORR 28%, DCR 94%, mPFS 5.8 mo; 10 mg/kg: ORR 43%	EpCAM	Active
NCT06243393	Sacituzumab Govitecan (SG)	II/III	–	Tumor response rate, Safety, PFS pending	Trop-2	Active
NCT05639156	T4H11-DM4	I	–	Safety, DLT, RP2D pending	DDR1	Active
NCT04622774	IMGC936	I	–	Safety, Pharmacokinetics, MTD pending	ADAM9	Active
NCT07106892	HLX43 (PD-L1 ADC)	II	60 (planned)	Primary endpoint: ORR by IRRC; Secondary: PFS, OS	PD-L1	Not yet recruiting
NCT05493683	Disitamab vedotin + Tislelizumab	II	29 (estimated)	Ongoing - Primary endpoint: ORR; Secondary: PFS, OS, DCR, DOR	HER2	Active
NCT05489211	Datopotamab deruxtecan (Dato-DXd)	II	582 (estimated)	Ongoing - TROPION-PanTumor03 (Substudy 5 for CRC)	TROP2	Active
NCT04410224	ASN004	I	19	Dose escalation completed - MTD determined	5T4	Completed
NCT04460456	SBT6050 + PD-1 inhibitor	I/Ib	58	FIH study - evaluating safety and efficacy	HER2	Active

ORR, objective response rate; mPFS, median progression-free survival; mOS, median overall survival; DCR, disease control rate; MTD, maximum tolerated dose; Q2W/Q3W, every 2/3 weeks; IHC, immunohistochemistry. Trastuzumab deruxtecan received FDA accelerated approval in 2024 for HER2-positive (IHC3^+^) solid tumors including colorectal cancer (tumor-agnostic indication), requiring prior systemic treatment failure. Trial status: Active (currently recruiting/treating), Active not recruiting (follow-up only), Completed (all data collection finished, published or not published), Terminated (prematurely discontinued). Data sourced from ClinicalTrials.gov and published literature as of Septembery 2025.

### 3.1 Targets on the surface of colorectal cancer cells

#### 3.1.1 HER2

HER2 amplification occurs in 3-5% of metastatic CRC, though its expression is limited compared to breast and gastric cancers. T-DXd (trastuzumab deruxtecan) has achieved clinical approval and demonstrates significant efficacy in HER2-positive CRC patients, representing a major therapeutic advance for this molecularly defined subgroup. Disitamab vedotin (RC48) has shown activation of the innate immune cGAS-STING pathway through antibody-mediated relief of HER2’s inhibitory effect on STING, producing type I interferons that enhance antigen presentation and promote cytotoxic T-cell infiltration (60, 61). Beyond T-DXd and RC48, key HER2-directed ADCs include A166 (trastuzumab-based; Val-Cit cleavable linker; Duostatin-5/auristatin; DAR≈2) (62), ZW49/zanidatamab zovodotin (biparatopic HER2; protease-cleavable; auristatin ZD02044; DAR≈2), SHR-A1811/trastuzumab rezetecan (camptothecin/Topoisomerase-I payload; DAR≈5.7), and SBT6050 (HER2-targeted TLR8 agonist; discontinued). Together they diversify payload class (microtubule vs Top1 vs immune-stimulatory) and seek better efficacy/safety in HER2^+^ CRC.

#### 3.1.2 CEACAM5

CEACAM5 is overexpressed in ~80-90% of CRC and associates with poorer outcomes. Tusamitamab ravtansine (DM4, a maytansinoid microtubule inhibitor) showed manageable safety in early studies but its global program was discontinued because an interim analysis of the Phase III CARMEN-LC03 trial failed to meet the primary endpoint (PFS). In contrast, Topoisomerase-I payloads appear more promising: labetuzumab govitecan (IMMU-130; SN-38, a topoisomerase I inhibitor) achieved a ~29% clinical benefit rate in heavily pretreated, irinotecan-refractory mCRC (63), and precemtabart tocentecan (M9140/Precem-TcT; exatecan; cleavable linker; high DAR ~8) has reported early disease control in refractory mCRC (64). Collectively, Top1-based CEACAM5 ADCs may offer stronger bystander effects than microtubule payloads, pending confirmation in randomized CRC trials. Next-generation concepts-bispecific CEACAM5/6 ADCs (e.g., CT109-SN-38) and single-domain (VHH) ADCs to boost tumor penetration-are advancing preclinically (65, 66).

#### 3.1.3 TROP2 (TACSTD2)

TROP2 is frequently expressed in CRC, with strong IHC positivity in about 20% (67, 68). Sacituzumab govitecan (SG) couples an anti-TROP2 antibody to SN-38 via the hydrolysable CL2A linker with a high DAR ~7.6, enabling membrane-diffusible bystander killing. In the IMMU-132–01 CRC cohort (n=31; heavily pretreated, many post-irinotecan), SG monotherapy achieved ORR 3.2%, median PFS 3.9 mo, and median OS 14.2 mo, suggesting cross-resistance. Still, TROP2 remains attractive: the TROPHIT-1 phase II/III trial is comparing SG vs SOC in refractory mCRC, and datopotamab deruxtecan (Dato-DXd)-a TROP2-DXd ADC with strong bystander effect-is being tested in CRC in TROPION-PanTumor03. Optimal benefit may require patient selection and rational combinations or earlier-line use.

#### 3.1.4 c-Met (HGFR)

c-Met is overexpressed in ~50% of CRC and mediates resistance to anti-EGFR/HER2 and KRAS G12C therapies. Telisotuzumab adizutecan (ABBV-400) links telisotuzumab (ABT-700) to the camptothecin-derived topoisomerase-I payload adizutecan via a cleavable linker (average DAR ≈6). In a first-in-human study, patients with high c-Met expression (IHC 3^+^ ≥10%) had an ORR of 37.5% at doses ≥2.4 mg/kg Q3W; lower-expressing tumors still showed responses (ORR 14%), consistent with bystander killing. Safety has been manageable so far, and randomized/combination cohorts (e.g., with 5-FU/leucovorin/bevacizumab) are underway. Overall, c-Met remains a compelling CRC target; Top1-payload ADCs may overcome intratumoral heterogeneity, pending confirmation in controlled trials.

#### 3.1.5 EpCAM (CD326)

EpCAM is broadly present on CRC but normal epithelial expression historically limited druggability. CX-2051 is a masked EpCAM ADC carrying a next-generation camptothecin Topo-I payload designed for tumor-local activation. Interim phase 1 data in heavily pretreated mCRC showed 28% ORR and 94% DCR across prioritized dose levels, 43% ORR at 10 mg/kg, and median PFS 5.8 months; most TRAEs were grade ≤2 and no DLTs were reported in escalation. A subsequent update noted a single grade 5 acute kidney injury in a patient with a solitary kidney; the safety committee supported study continuation with monitoring. CX-2051 illustrates a viable, tumor-selective way to “drug” EpCAM in CRC; expansion cohorts will clarify durability, dose, and risk mitigation.

#### 3.1.6 CEACAM6

CEACAM6 exhibits differential expression across CRC molecular subtypes, with highest levels in CMS4 tumors characterized by stromal infiltration and poor prognosis. The preclinical ADC 84-EBET demonstrated complete tumor regression in CRC PDX models. Notably, combination with PD-1 blockade enhanced CD8^+^ T cell infiltration, suggesting potential to overcome immune resistance in stroma-rich tumors (69). While preclinical results are encouraging for CMS4 subtype targeting, clinical validation is needed to confirm therapeutic benefit.

#### 3.1.7 HER3

HER3 (ERBB3) is often upregulated in CRC, though its impaired kinase activity has made it challenging for direct inhibition. U3-1402 (patritumab deruxtecan) combines anti-HER3 antibody with topoisomerase I inhibitor DXd via a cleavable linker (DAR≈8). Topoisomerase I inhibitors like DXd are recognized as powerful ICD inducers through catastrophic DNA damage (70). The membrane-permeable DXd payload enables potent bystander effects, killing adjacent HER3-negative tumor cells and potentially remodeling the local tumor milieu. Preclinical studies demonstrated significant tumor inhibition and complete responses in HER3-high CRC xenografts irrespective of KRAS mutation status, transforming an “undruggable” target into a conduit for delivering immunomodulatory payload. This strategy offers a promising therapeutic avenue for CRC, including tumors resistant to conventional EGFR-targeted therapies.

#### 3.1.8 PDL1

Programmed death-ligand 1 (PD-L1) is an immune checkpoint protein overexpressed on tumor cells, including CRC. Liu et al. (2023) developed a modular platform using poly (glutamic acid) scaffolds for noncovalent Fc-domain conjugation, generating anti-PD-L1 conjugates (aPDL1-P-MMAE, DAR = 40.7) achieving 98.5% tumor growth inhibition in MC38 CRC models without systemic toxicity (71). Zhang et al. (2023) extended this to aPDL1-NPLG-SN38 (DAR = 72) with 2.8-fold higher tumor accumulation versus non-targeted IgG conjugates, demonstrating excellent therapeutic properties in both medium-sized and large MC38 tumor models (72). These studies offer a promising platform for designing ultrahigh-DAR ADCs with preserved antigen-binding capacity, integrating chemical innovation, immune modulation, and high-precision drug delivery.

#### 3.1.9 CD47

CD47 functions as a “don’t eat me” signal by binding SIRPα on myeloid cells, enabling immune evasion. Chiang et al. (2024) developed non-cleavable CD47-targeting ADCs (7DC2-DM1, 7DC4-DM1) showing near-complete tumor inhibition in CRC and lung cancer models with improved safety versus cleavable constructs (73). This strategy not only blocks the CD47-SIRPα axis to enhance macrophage phagocytosis, but also delivers DM1 for direct tumor killing that releases tumor antigens for immune presentation. CD47-targeted ADCs represent an elegant dual-mechanism approach to convert immunologically “cold” tumors into “hot” ones.

#### 3.1.10 GCC

Guanylyl Cyclase C (GCC) shows exceptional tumor specificity in CRC, being almost exclusively restricted to intestinal cells, with expression in 98% of primary CRC and in ≥95% of metastatic lesions. TAK-164, carrying the DNA-alkylating payload DGN549, demonstrated strong activity in PDX models (74). The Phase I trial confirmed on-target DNA damage (elevated γH2AX) in patient biopsies (75). However, dose-limiting hepatotoxicity (including grade 5 hepatic failure) led to trial termination, with the tolerable dose (0.064 mg/kg) deemed insufficient for clinical benefit while higher doses (≥0.19 mg/kg) proved too toxic. This highlights the critical challenge of achieving adequate therapeutic window.

#### 3.1.11 DDR1

Discoidin domain receptor 1 (DDR1) is overexpressed in >80% of CRC and linked to poor prognosis and chemoresistance. T4H11-DM4 (maytansinoid microtubule inhibitor) achieved complete tumor regression in oxaliplatin-resistant xenograft models (76). The DM4 payload induces mitotic arrest and apoptosis. By targeting a resistance-associated antigen, this strategy offers direct killing of treatment-refractory cells. The ADC shows promise for addressing chemoresistant CRC populations, though further studies are needed to characterize its full therapeutic potential.

#### 3.1.12 DR5

Death receptor 5 (DR5) is overexpressed in CRC. Oba01 links anti-DR5 antibody zaptuzumab to MMAE (a microtubule inhibitor), creating dual mechanisms: apoptosis via DR5 signaling and cytotoxicity via MMAE (77) Preclinical studies in pancreatic cancer models demonstrated anti-tumor activity. Further research is needed to evaluate this approach specifically in CRC, particularly in treatment-refractory populations.

#### 3.1.13 ADAM9

ADAM9 is a transmembrane metalloproteinase overexpressed in CRC with minimal normal tissue expression. IMGC936, site-specifically conjugated to maytansinoid DM21C (a microtubule inhibitor), achieved complete tumor regression in xenograft models and entered Phase I trials (78). The ADC demonstrated potent direct cytotoxicity and bystander killing effects in preclinical studies. However, clinical development was discontinued after failing to meet pre-established clinical safety and efficacy benchmarks in Phase 1, highlighting the challenges of translating preclinical efficacy to clinical benefit.

#### 3.1.14 DOG1

DOG1 (Discovered on GIST1) exhibits tumor-restricted expression in CRC with high mRNA positivity and high expression in liver metastases. An anti-DOG1-DM4 ADC showed potent activity in CRC liver-metastasis model (79). The ADC shows efficacy at metastatic sites while maintaining preserved liver function. This approach is particularly relevant for addressing the challenge of hepatic metastases in CRC, a common site of disease progression.

#### 3.1.15 CD228

CD228 (melanotransferrin) is a GPI-anchored membrane protein with minimal expression in normal tissues and elevated expression in multiple solid tumors, including CRC. SGN-CD228A links humanized anti-CD228 antibody hL49 to MMAE via a PEGylated glucuronide linker (80). The IgG1 backbone is capable of mediating ADCC, while controlled intracellular MMAE release triggers potent cytotoxicity and stimulates DAMP release. These signals act as an endogenous vaccine, recruiting and activating antigen-presenting cells to prime a T-cell-mediated anti-tumor immune response.

#### 3.1.16 EGFR

EGFR is frequently overexpressed in 60-80% of CRC, including KRAS-mutant disease where conventional EGFR inhibitors fail. Novel EGFR-targeted ADCs aim to overcome this resistance through immunomodulatory mechanisms (81). Bisphosphonate-conjugated ADC cetuximab-zoledronate (Cet-ZA) demonstrated direct cytotoxicity plus activation of Vγ9Vδ2 T cells in CRC organoid models, potentially bridging targeted therapy with innate immunity (82). The IgG1 backbone inherently mediates ADCC via NK cell recruitment. While these immunoconjugates remain in preclinical development (83). they represent a rational strategy to transform EGFR into an immune-activating platform for KRAS-mutant and treatment-refractory CRC.

### 3.2 Targets on the surface of colorectal cancer stem cells

#### 3.2.1 LGR5

Leucine-rich repeat-containing G protein-coupled receptor 5 (LGR5) is a definitive marker for both normal intestinal stem cells and colorectal CSCs, playing fundamental roles in tumor initiation and progression via WNT signaling (84, 85). This shared expression creates significant therapeutic window challenges-the primary barrier to clinical translation. Early preclinical ADC studies showed highly potent payloads could induce severe on-target, off-tumor toxicity in normal LGR5-expressing tissues despite anti-tumor efficacy, establishing that therapeutic index is critical. To overcome this, petosemtamab (MCLA-158) was developed as a bispecific antibody targeting both LGR5 and EGFR, leveraging EGFR co-expression to selectively target tumors while sparing healthy LGR5+ intestinal stem cells (86) Petosemtamab employs EGFR degradation and enhanced immune-mediated destruction through ADCC and ADCP (87). After demonstrating superior efficacy over standard EGFR inhibitors in patient-derived organoids and xenografts, it is now in clinical trials for metastatic CRC.

#### 3.2.2 CD133

CD133 is a five-domain transmembrane glycoprotein and a well-established surface marker of CSCs in CRC. Its expression is closely associated with tumor initiation, metastasis, therapy resistance, and recurrence. Given the central role of CD133^+^ cells in treatment failure, targeting them represents a critical strategy for preventing disease progression. Preclinical studies explored nanocarrier systems delivering conventional chemotherapeutics like oxaliplatin and 5-FU directly to CD133^+^ cells-both are known ICD inducers. By forcing immunogenic cell death, these agents trigger DAMP release, recruiting and activating antigen-presenting cells (88, 89). While these systems remain in preclinical development, they highlight a promising approach to overcome chemoresistance and potentially reverse immune ignorance by targeting the CSC.

#### 3.2.3 GPR56

G protein-coupled receptor 56 (GPR56) (ADGRG1) overexpression in CRC correlates with poor survival and increased postoperative relapse, with particular enrichment in microsatellite stable (MSS) disease-the predominant immune checkpoint inhibitor (ICI)-refractory subtype (90, 91). Mechanistically, GPR56 activates the RhoA-MDR1 signaling axis to enhance efflux-mediated chemoresistance (92) and maintains LGR5-negative stem-like cells in a treatment-refractory state. A GPR56-targeted antibody-drug conjugate (ADC) in preclinical study utilizing duocarmycin SA, a DNA minor-groove alkylating agent, demonstrated target-dependent tumor growth inhibition in CRC xenografts and patient-derived organoids with acceptable tolerability (90, 93).

#### 3.2.4 5T4

5T4 is an oncofetal glycoprotein (72kDa trophoblast cell surface antigen) that is minimally expressed in adult normal tissues but is overexpressed in a wide range of malignancies, including colorectal cancer. In CRC, 5T4 is associated with tumor invasiveness and stem-like features, making it an appealing target to eliminate aggressive cancer cell subpopulations. ASN004 (scFv-Fc format) uses Dolaflexin polymer to deliver auristatin F-HPA (microtubule inhibitor) at very high DAR (~10-12), achieving deep regressions preclinically; first-in-human studies report manageable safety, with efficacy readouts pending.

#### 3.2.5 Epiregulin

Epiregulin (EREG), a ligand of the EGFR family, is aberrantly upregulated in a substantial subset of CRC, including both RAS wild-type and mutant subtypes. Its expression in both differentiated tumor cells and undifferentiated cancer stem-like populations suggests a role in tumor plasticity and therapy resistance.Based on Jacob et al.’s preclinical study, a humanized anti-EREG antibody (H231) conjugated to duocarmycin DM via enzymatically cleavable tripeptide linkers was developed (94, 95). Their lead candidate, H231 EGC-qDuoDM gluc, demonstrated subnanomolar potency in EREG-expressing CRC cells irrespective of RAS status and achieved significant tumor growth inhibition in both cell line xenografts (70% TGI in LoVo, 68% in DLD-1) and patient-derived xenografts (86-88% TGI in MSS models). Notably, the ADC outperformed cetuximab and showed acceptable tolerability in immunocompetent mice. Future development should prioritize comprehensive pharmacokinetic/pharmacodynamic studies, formal assessment of immunogenic cell death markers (HMGB1, calreticulin, ATP release), and evaluation of combination strategies with immune checkpoint inhibitors in syngeneic models to fully realize the immunotherapeutic potential of this promising EREG-targeted approach.

## 4 Clinical evidence and CRC-specific challenges

### 4.1 Trials landscape & key signals in CRC

The clinical landscape of CRC treatment has evolved from conventional chemotherapy and targeted antibodies to ADCs. Traditional chemotherapy (5-FU, irinotecan, oxaliplatin) lacks selectivity, causing systemic toxicity and immunosuppression despite tumor-agnostic efficacy (96). Monoclonal antibodies (cetuximab, bevacizumab) offer specificity but limited direct cytotoxicity, relying on pathway inhibition vulnerable to resistance mechanisms. ADCs uniquely combine chemotherapy’s potency with antibody selectivity, delivering ultra-potent payloads specifically to antigen-expressing cells while sparing normal tissues. The bystander effect enables ADCs with membrane-permeable payloads to kill neighboring antigen-negative populations through local drug diffusion, partially compensating for tumor heterogeneity. Additionally, select ADC payloads induce immunogenic cell death, recruiting T-cells and converting “cold” MSS tumors to “hot” phenotypes, creating immune engagement absent in conventional therapies.

The clinical development of ADCs in colorectal cancer demonstrates distinct patterns of success and failure, fundamentally determined by payload selection. **Table 1** summarizes ongoing and completed clinical trials. Among these, trastuzumab deruxtecan (T-DXd), carrying a topoisomerase I inhibitor payload, received FDA approval for unresectable or metastatic HER2-positive (IHC3^+^) solid tumors. In the DESTINY-CRC01 trial, T-DXd achieved an objective response rate (ORR) of 45.3% in HER2-positive patients, reaching 57.5% in the IHC3^+^ subgroup, validating the importance of appropriate payload-tumor matching. This success contrasts sharply with the consistent failure of microtubule inhibitor-based ADCs. TAK-264 (anti-GCC-MMAE), despite targeting an antigen expressed in >90% of CRCs, demonstrated zero clinical responses, likely reflecting intrinsic resistance of colorectal tumors to microtubule inhibitors. The payload-specificity issue is further exemplified by T-DM1’s differential efficacy across tumor types. While T-DM1 significantly improves progression-free and overall survival in HER2-positive breast cancer, its efficacy in HER2-positive CRC remains minimal, with only one responder among eight patients (97). These findings underscore that successful ADC development requires payload selection tailored to tumor-specific biological characteristics rather than simply matching target expression profiles.

Clinical translation of ADCs in CRC remains nascent despite platform maturation. With ~15 approved ADCs across solid tumors, application in CRC lags behind breast and gastric cancers, primarily due to limited validated targets and desmoplastic barriers. Current clinical investigations focus on CEACAM5, HER2, and Trop-2, yet antigen heterogeneity and stromal density pose formidable obstacles.

### 4.2 CRC-specific obstacles

Despite successful ADC approvals in multiple malignancies, including the recent tumor-agnostic approval of trastuzumab deruxtecan for HER2-positive solid tumors, only T-DXd has achieved regulatory approval specifically for colorectal cancer. This limited success reflects CRC-specific biological barriers that impede ADC efficacy.

First, target antigen heterogeneity presents a fundamental challenge. HER2 amplification occurs in merely 2-5% of metastatic CRCs (98), while even prevalent targets like CEACAM5 display intratumoral heterogeneity, with antigen-negative cells interspersed among positive populations (99, 100). Second, inefficient internalization limits payload delivery. Certain CRC-associated antigens, particularly CEACAM5, demonstrate slow internalization kinetics upon antibody binding, reducing intracellular drug accumulation (101). Third, the physical tumor microenvironment creates formidable delivery barriers. Dense desmoplastic stroma and elevated interstitial fluid pressure impede antibody penetration, resulting in heterogeneous intratumoral ADC distribution. Their 150-kDa size limits penetration in desmoplastic CRC tumors, potentially creating sanctuary sites.Fourth, immunological exclusion in MSS tumors diminishes therapeutic response. Approximately 95% of CRCs are microsatellite stable, characterized by “cold” immune microenvironments lacking cytotoxic T-cell infiltration. This immune exclusion eliminates potential contributions from antibody-dependent cellular cytotoxicity and immunogenic cell death following ADC treatment.

Additionally, on-target/off-tumor gastrointestinal toxicity remains problematic. Many candidate antigens, including Trop-2, exhibit baseline expression in normal intestinal epithelium, causing dose-limiting gastrointestinal adverse events that narrow the therapeutic window. The therapeutic window narrows further as systemic toxicities—neutropenia, ocular damage, thrombocytopenia—constrain dosing (102). These CRC-specific challenges collectively explain the limited clinical translation of ADCs in this malignancy despite successes elsewhere. Future success hinges on identifying CRC-enriched antigens, particularly on TME components (CAFs, CSCs) and leveraging payloads with robust bystander effects to overcome spatial heterogeneity (103).

#### 4.2.1 Antigen heterogeneity & expression threshold

ADC efficacy critically depends on target antigen expression levels and distribution uniformity. Quantitative studies suggest approximately 10,000 receptors/cell as a functional threshold for effective ADC activity (104). The DESTINY-CRC01 trial exemplified this principle, achieving 45.3% objective response rate in HER2 IHC3^+^/ISH^+^ patients while observing no responses in HER2-low cohorts, highlighting the importance of stringent expression criteria. Intratumoral heterogeneity poses additional challenges, with antigen-negative clones interspersed among positive populations. The bystander effect offers a potential solution: hydrophobic payloads like DXd and SN-38 can diffuse from antigen-positive cells to eliminate neighboring antigen-negative cells. Emerging strategies include bispecific ADCs targeting dual epitopes to enhance receptor clustering and internalization. MEDI4276, binding two HER2 epitopes simultaneously, demonstrated accelerated lysosomal trafficking preclinically, though early-phase trials revealed narrow therapeutic windows. Optimized patient selection through IHC scoring, H-score thresholds, and RNA-based quantification may help identify optimal candidates. These approaches collectively suggest pathways to mitigate heterogeneity-related limitations in CRC ADC development.

#### 4.2.2 Internalization & intracellular trafficking kinetics

Efficient ADC activity requires optimal antigen-antibody complex internalization and lysosomal trafficking. In colorectal cancer, internalization kinetics vary significantly among target antigens. CEACAM5, despite widespread expression in CRC, demonstrates notably slow internalization upon antibody binding, limiting intracellular payload delivery. This contrasts with other targets being evaluated in CRC clinical trials. Epitope selection proves crucial-dual-epitope targeting can induce receptor clustering and accelerate endocytosis, as demonstrated in preclinical HER2-targeting studies. Linker design significantly impacts payload release dynamics: cleavable linkers enable faster intracellular drug liberation but risk premature systemic release, while non-cleavable linkers require complete antibody degradation (105). Novel tumor-microenvironment-activated linkers represent an emerging strategy to maintain circulation stability while facilitating tumor-specific activation, though CRC-specific applications await clinical validation (105). Quantitative studies across multiple tumor types suggest compensatory relationships between antigen density and internalization rates. For CRC-relevant targets like CEACAM5, HER2, and GCC, optimizing these parameters during ADC design may help overcome the internalization barriers specific to colorectal tumors.

#### 4.2.3 Tumor penetration limits & physical TME barriers

Colorectal tumors present formidable physical barriers impeding ADC distribution. The 150-kDa antibody size restricts diffusion through dense extracellular matrix, while irregular vasculature and elevated interstitial pressure further limit penetration (106). The “binding-site barrier” phenomenon-whereby ADCs saturate perivascular antigens before reaching deeper tumor regions-compounds distribution challenges. ADC dosing constraints, necessitated by payload toxicity, may result in subtherapeutic concentrations in poorly perfused areas (107). Several strategies show promise for enhancing penetration. Bystander-effect payloads enable killing of antigen-negative or inaccessible cells through local diffusion. Smaller antibody formats (scFv, Fab fragments, nanobodies) demonstrate improved tissue penetration, albeit with faster clearance (108). Co-administration of unlabeled carrier antibodies may saturate peripheral binding sites, driving deeper ADC penetration. The desmoplastic CRC microenvironment, characterized by fibrotic stroma and hypoxic regions, creates heterogeneous drug distribution patterns. These physical barriers likely contribute to treatment resistance and warrant continued investigation of penetration-enhancing strategies tailored to CRC-specific microenvironmental features.

#### 4.2.4 Immunological dichotomy between MSS and MSI-H colorectal cancer

Microsatellite status substantially influences CRC immunobiology and therapeutic responses. MSI-H tumors (15% of cases) typically exhibit high mutational burden, abundant neoantigens, and robust T-cell infiltration, contributing to their responsiveness to checkpoint inhibitors (109, 110) In contrast, MSS tumors (85% of cases) generally display low mutational burden, minimal neoantigen presentation, and “cold” microenvironments characterized by sparse T-cell infiltration and abundant immunosuppressive cells (111–113). This dichotomy significantly impacts treatment outcomes: while MSI-H patients often achieve 40-60% response rates with checkpoint inhibitors, MSS patients show limited benefit (114, 115). ADCs may provide valuable opportunities for MSS CRC treatment (116). Cytotoxic payloads could potentially induce immunogenic cell death, possibly converting “cold” tumors to “hot” phenotypes. Zhou et al. developed Oba01, a DR5-targeting ADC conjugated with MMAE via cleavable linker (77), representing efforts to address MSS CRC challenges. Strategic payload selection favoring immunogenic mechanisms, combined with immune checkpoint blockade, may help mitigate the immunosuppressive MSS microenvironment, though clinical validation remains essential.

### 4.3 Resistance mechanisms (intrinsic & acquired; payload-specific)

ADC resistance involves multifaceted mechanisms spanning both intrinsic and acquired pathways. Intrinsic resistance often stems from pre-existing cellular features. ABC transporter overexpression, particularly P-glycoprotein, promotes drug efflux and reduces intracellular accumulation (117). MMAE-based ADCs face notable challenges in gastrointestinal cancers, where P-gp expression frequently increases following chemotherapy exposure (118, 119). This may partially explain the failure of TAK-264 (anti-GCC-MMAE) in colorectal cancer trials despite high GCC expression (120, 121). Emerging strategies include selecting efflux-insensitive payloads like topoisomerase I inhibitors, as demonstrated by T-DXd’s success in DESTINY-CRC01, contrasting with MMAE-based failures (122).

Acquired resistance develops dynamically under treatment pressure through multiple mechanisms. Target antigen modulation represents a primary escape route-downregulation, mutation, or selection of antigen-negative clones can emerge within months of treatment initiation. In tumors with heterogeneous antigen expression like CEACAM5 in colorectal cancer, pre-existing low-expressing populations may expand under selection pressure. Payload-specific resistance patterns vary: topoisomerase I inhibitor resistance involves TOP1 downregulation, enhanced DNA repair pathway activation, and apoptosis evasion through NF-κB activation. MMAE resistance primarily involves efflux pump upregulation and tubulin alterations. Compensatory signaling pathways provide additional escape mechanisms-alternative receptor tyrosine kinases may maintain downstream signaling despite target blockade. These multilevel resistance mechanisms suggest combination approaches may prove valuable for sustained efficacy.

ADCs also remain constrained by antigen dependency—tumors lacking suitable targets escape treatment. Acquired resistance through antigen loss or downregulation parallels targeted therapy resistance patterns. Unlike chemotherapy’s antigen-independent activity, ADCs require sustained target expression for efficacy.

### 4.4 Safety profile in CRC & mitigation (GI/hematologic/ILD)

ADC-related toxicities in colorectal cancer trials encompass both on-target and off-target effects, with gastrointestinal and hematologic adverse events predominating. In DESTINY-CRC01, trastuzumab deruxtecan demonstrated near-universal adverse event occurrence, with grade ≥3 neutropenia (22.1%) and anemia (14.0%) most frequently observed (123, 124). Gastrointestinal toxicities, including nausea, diarrhea, and mucositis, likely result from topoisomerase I inhibitor payloads (SN-38, DXd) directly affecting intestinal epithelium and hepatobiliary excretion of free toxins (125). IMMU-130 (labetuzumab govitecan) similarly showed manageable toxicity profiles with grade ≥3 neutropenia (16%), leukopenia (11%), and diarrhea (7%) as dose-limiting toxicities.

Interstitial lung disease represents a particularly concerning ADC-specific toxicity. T-DXd trials reported ILD/pneumonitis in 9.3% of CRC patients, including fatal cases (3.5%), necessitating careful patient selection and monitoring (126). Dose optimization has proven effective-reducing T-DXd from 6.4 to 5.4 mg/kg in DESTINY-CRC02 eliminated grade 5 toxicities while maintaining efficacy (127).

Mitigation strategies focus on multiple approaches. Linker optimization enhances stability to minimize premature payload release, as demonstrated by YL201’s hydrophilic linker achieving only 1.3% severe ILD incidence. Dose fractionation reduces peak concentration-related toxicities-IMMU-130’s weekly dosing showed improved tolerability versus every-three-week schedules. Supportive care measures include prophylactic G-CSF for anticipated neutropenia, early antidiarrheal intervention for SN-38-based ADCs, and antiemetic premedication. Careful monitoring protocols enable timely dose modifications: most hematologic toxicities resolve within 1–2 weeks of treatment interruption, allowing dose-reduced continuation (128). Through these integrated management strategies, approximately 85% of patients complete intended therapy despite high adverse event rates, suggesting ADC toxicities remain manageable within appropriate frameworks.

## 5 Discussion and conclusion

ADCs are rapidly transitioning from targeted chemotherapies into a sophisticated class of immuno-oncology agents, representing a new frontier for CRC treatment. While preclinical studies have shown remarkable promise, the journey to clinical approval remains challenging. The future success of ADCs in CRC hinges on a paradigm shift: moving beyond direct cytotoxicity to strategically harnessing their profound ability to modulate the TME and synergize with the host immune system. The true innovation in next-generation ADCs lies in the immunological consequences of their payload selection.

This immunotherapeutic lens also redefines what constitutes an optimal target. The focus is expanding from antigens on tumor cells (CEACAM5, HER2) to include those on critical TME components, such as cancer-associated fibroblasts (CEACAM6), or on immune checkpoints themselves (PD-L1, CD47). Targeting CSCs with markers like LGR5 or GPR56 using an ICD-inducing ADC is a particularly powerful strategy, as it aims to eradicate the root of relapse while simultaneously initiating an immune response from the most resilient tumor population. This dual-pronged attack-debulking the tumor while disabling its defenses-is central to the modern ADC concept.

Immune priming mechanisms position ADCs as ideal immuno-oncology partners. ADC-mediated ICD releases tumor antigens, activates cGAS-STING signaling, and recruits cytotoxic T-cells, converting immunologically “cold” MSS tumors to “hot” phenotypes (102). Immunostimulatory payloads exemplify this paradigm: photoimmunotherapy platforms induce dendritic cell maturation and amplify CD8^+^ responses via localized ICD (103). This immune activation creates synergy with checkpoint inhibitors, as demonstrated in preclinical models where ADC pretreatment enhances anti-PD-1 efficacy (129). The dual capacity for direct cytotoxicity and immune engagement distinguishes ADCs from conventional targeted therapies. The antibody component further enables Fc-mediated ADCC and phagocytosis, creating synergistic immune engagement that maintains efficacy despite variable antigen expression.

Biomarker-driven patient selection and rational combination strategies remain critical. Current approaches relying on binary antigen expression are insufficient; integration of antigen density, spatial uniformity, and immune contexture (GSDME expression, cGAS-STING activity) is essential for optimizing patient selection. Payload selection must balance potency, membrane permeability for bystander killing, and hydrophilicity to minimize off-target toxicity while maintaining DAR and linker stability (103). Despite third/fourth-generation engineering advances (102), translational barriers persist, necessitating systematic evaluation of ADC-immunotherapy combinations in biomarker-stratified clinical trials to fully unlock their therapeutic potential in CRC.

To realize this vision, biomarker development must evolve beyond simple antigen expression. The selection of patients for ADC therapy should incorporate immuno-profiling to assess the TME, expression of key cell death mediators like GSDME, or activation of pathways such as cGAS-STING. In conclusion, ADCs offer a modular and mechanistically versatile platform with the potential to reshape the CRC treatment landscape. Their ultimate success will be driven by continued innovation in molecular engineering and, most critically, by their intelligent integration into biomarker-driven, immuno-oncology combination strategies designed to kill tumor cells and awaken the immune system in a single, coordinated assault.
